# Meta-Analysis Reveals Significant Sex Differences in Chronic Lymphocytic Leukemia Progression in the *Eµ-TCL1* Transgenic Mouse Model

**DOI:** 10.3390/cancers12071980

**Published:** 2020-07-20

**Authors:** Maximilian Koch, Sebastian Reinartz, Julia Saggau, Gero Knittel, Natascha Rosen, Oleg Fedorchenko, Lisa Thelen, Romy Barthel, Nina Reinart, Tamina Seeger-Nukpezah, Hans Christian Reinhardt, Michael Hallek, Phuong-Hien Nguyen

**Affiliations:** 1 University of Cologne, Department I of Internal Medicine, Center for Integrated Oncology Aachen Bonn Cologne Duesseldorf, Center for Molecular Medicine Cologne, CECAD Center of Excellence on Cellular Stress Responses in Aging-Associated Diseases, 50931 Cologne, Germany; maximilian.koch@uk-koeln.de (M.K.); sebastian.reinartz@uk-koeln.de (S.R.); julia.saggau@uk-koeln.de (J.S.); gero.knittel@uk-koeln.de (G.K.); natascha.rosen@uk-koeln.de (N.R.); oleg.fedorchenko@uk-koeln.de (O.F.); lisa.thelen@uk-koeln.de (L.T.); romy.barthel@uk-essen.de (R.B.); nina.reinart@googlemail.com (N.R.); tamina.seeger-nukpezah@uk-koeln.de (T.S.-N.); christian.reinhardt@uk-koeln.de (H.C.R.); michael.hallek@uni-koeln.de (M.H.); 2 Clinic for Hematology, West German Cancer Center, University Hospital Essen, Essen, German Cancer Consortium (DKTK), 45147 Essen, Germany

**Keywords:** chronic lymphocytic leukemia, sex difference, *TCL1*, transgenic mouse model, adoptive transplantation

## Abstract

The *Eµ-TCL1* transgenic mouse model represents the most widely and extensively used animal model for chronic lymphocytic leukemia (CLL). In this report, we performed a meta-analysis of leukemia progression in over 300 individual *Eµ-TCL1* transgenic mice and discovered a significantly accelerated disease progression in females compared to males. This difference is also reflected in an aggressive CLL mouse model with additional deletion of *Tp53* besides the *TCL1* transgene. Moreover, after serial adoptive transplantation of murine CLL cells, female recipients also succumbed to CLL earlier than male recipients. This sex-related disparity in the murine models is markedly contradictory to the human CLL condition. Thus, due to our observation we urge both careful consideration in the experimental design and accurate description of the *Eµ-TCL1* transgenic cohorts in future studies.

## 1. Introduction

Increasing understanding of the biology and pathogenesis of chronic lymphocytic leukemia (CLL) has led to many breakthroughs in the treatment of this disease [1,2], which has been acquired owing considerably to the use of animal models. To date, the *Eµ-TCL1* transgenic mouse model is the most widely used CLL model, indicated by over 500 citations of the original paper [3]. The ectopic expression of the human T cell leukemia 1 (*TCL1*) oncogene under the control of the V_H_-promoter and Ig_H_-Eµ-enhancer in transgenic mice enables the development of a highly similar CLL-like disease with 100% penetrance. The close resemblance of the human CLL disease and high penetrance in the *Eµ-TCL1* transgenic mice renders this model a popular tool to study pathogenic interactions leading to CLL. The *Eµ-TCL1* transgenic mice have been extensively used in the field of CLL research, in which these mice either were intercrossed with a vast variety of mice bearing other mutations or served as a pre-clinical model for different treatment options [4,5,6,7]. Given their intensive usage, a thorough understanding of CLL pathogenesis in *Eµ-TCL1* transgenic mice is critical for the precise interpretation of the acquired results.

The prevalent influences of sex on the disease phenotypes of many mouse lines have been evaluated [8], including several mouse models for cancers [9,10,11]. Although the incidence and clinical course of CLL is strongly different between men and women [1], the sex-related characteristics of the CLL mouse model remain unexplored. Here, we report a profound difference in the leukemic progression and overall survival between male and female *Eµ-TCL1* transgenic mice, which is markedly contradictory to the human CLL condition. Our finding based on the analysis of over 300 mice argues for unbiased experiment design and more accurate description of the *Eµ-TCL1* transgenic mouse cohorts in future studies.

## 2. Materials and Methods

### 2.1. Mouse Cohorts and Housing Facilities

All mouse experiments were approved by the state authorities of North Rhine–Westphalia, Germany (LANUV) #K17,12/04; #9.93.2.10.31.07.097; #9.93.2.10.31.07.098; #8.87-50.10.37.09.241; #84-02.04.2014.A146; #84-02.04.2016.A058. All analyzed mice were hemizygote for the *TCL1* transgene (*Eµ-TCL1^tg/wt^*), had either a hybrid C3H/HeJ × C57BL/6 (B6C3) or a C57BL/6 (B6) genetic background, and were housed in groups of up to five animals per cage in individually ventilated cages (IVC) in three different animal facilities of the University Hospital of Cologne (Table 1). Mice housed in the Institute of Experimental Medicine (EM) and the Institute of Pathology (PA) were specific pathogen-free (SPF), mice housed in the facility of the CECAD Research Center were specific and opportunistic pathogen-free (SOPF). Mice from four cohorts harbored additional, unaffecting mutations besides the *TCL1* transgene (Table 1).

### 2.2. Mouse Blood Analyses

Procedures for differential blood counts and determination of CLL burden were previously described [12,13,14,15]. Leukocyte count (LC) was measured with a SYSMEX XE-5000 system (Sysmex, Kobe, Japan). CLL cells defined as CD19^+^ CD5^+^ or IgM^+^ CD5^+^ were identified by flow cytometry with either a BD FACSCanto (BD, Franklin Lakes, New Jersey), or a Gallios (Beckman Coulter, Brea, CA, USA), or a MACSQuant *VYB* or a MACSQuant *X* (Miltenyi Biotec, Bergisch Gladbach, Germany). Data were analyzed using Kaluza Flow Analysis Software (Beckman Coulter) or FlowJo™ Analysis Software (BD).

### 2.3. Survival Determination

Mice reaching endpoint defined by the animal welfare law were euthanized by cervical dislocation. All death events unrelated to leukemia were excluded from this study. Survival of *Eµ-TCL1* transgenic mice was recorded from birth until death or euthanasia. Survival of transplanted recipients was recorded from the day of CLL injection until death or euthanasia.

### 2.4. Syngeneic Adoptive Transplantation

Freshly homogenized and filtered splenocytes from moribund *Eµ-TCL1* transgenic mice of pure C57BL/6 (J) genetic background were layered with Pancoll separating solution (PAN Biotech, Aidenbach, Germany), followed by centrifugation and separation of interphase-concentrated mononuclear cells. After several washing steps, 10^7^ cells were injected intraperitoneally into sex-matched C57BL/6 (J) mice between eight and 14 weeks of age, generating Passage 1 (P1) recipients. Similar procedures were applied in Passage 2 (P2) transplantation, in which splenocytes of moribund P1 recipients were injected into P2 recipients.

### 2.5. Statistical Analysis

All statistical differences of blood data were calculated with the Mann–Whitney test, comparisons of the survival curves were calculated with the Mantel–Cox logrank test using Prism 8 (GraphPad Software, San Diego, CA, USA).

## 3. Results

**Figure 1:**
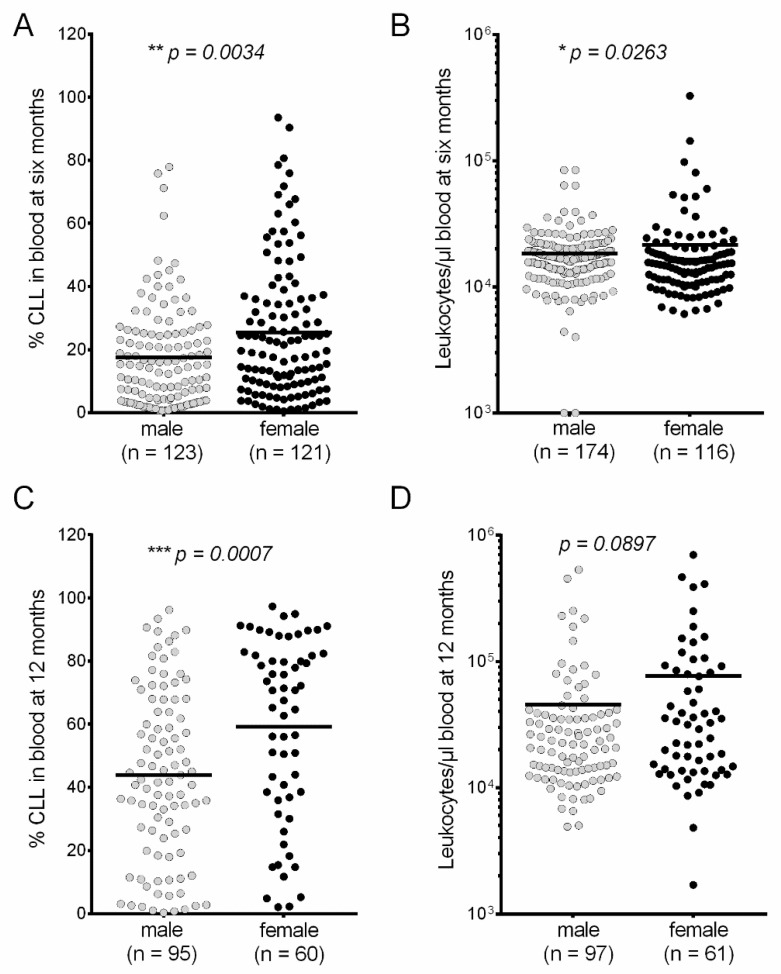
Significant sex difference in chronic lymphocytic leukemia (CLL) progression in the *Eµ-TCL1^tg/wt^* mice. (**A**) Significantly lower CLL load in the peripheral blood of males compared to females at six months. Blood samples from tail vein were assessed by flow cytometry to determine the CD5-positive CLL percentage in lymphocytes. Males: *n* = 123; Mean ± SEM: 17.55% ± 1.435%. Females: *n* = 121; Mean ± SEM: 25.43% ± 1.953%. Mann–Whitney test: ** *p* = 0.0034. (**B**) Lower leukocyte count in the peripheral blood (cells per µL) in male than female *Eµ-TCL1^tg/wt^* mice at six months. Males: *n* = 174; Mean ± SEM: 18,420 ± 821.3; Females: *n* = 116; Mean ± SEM: 21,556 ± 3112; Mann–Whitney test: * *p* = 0.0263. (**C**) Significantly lower CLL load in the peripheral blood of males compared to females at 12 months. Males: *n* = 95; Mean ± SEM: 43.87% ± 2.758%. Females: *n* = 60; Mean ± SEM: 59.24% ± 3.701%. Mann–Whitney test: *** *p* = 0.0007. (**D**) Lower leukocyte count in the peripheral blood (cells per µL) in male than female *Eµ-TCL1^tg/wt^* mice at 12 months. Males: *n* = 97; Mean ± SEM: 45,581 ± 8,089; Females: *n* = 61; Mean ± SEM: 76,442 ± 16,062; Mann–Whitney test: *p* = 0.0897.

We assessed CLL progression in male and female *Eµ-TCL1* transgenic mice in 11 independent studies ([12,13,14,15] and unpublished data) regarding the percentage of CD5 positive B-CLL cells in the murine peripheral blood (% CLL) at six and 12 months, and the overall survival (OS). Our meta-analysis of all *Eµ-TCL1^tg/wt^* mice revealed a significantly slower CLL progression in males compared to their female counterparts. At six months—the estimated time of disease establishment—% CLL was significantly lower by 7.876% in males versus females (Males: *n* = 123; Mean ± SEM: 17.55% ± 1.435%. Females: *n* = 121; Mean ± SEM: 25.24% ± 1.953%) (Figure 1A). At this time point, the mean leukocyte count in blood (LC) was slightly lower in males compared to females by 3,136 cells/µL (Males: *n* = 174; Mean ± EM: 18,420 ± 821.3. Females: *n* = 116; Mean ± SEM: 21,556 ± 3112) (Figure 1B). The difference in CLL burden became more compelling at the fully developed disease state of 12 months, the sex difference in % CLL increased to 15.37% (Males: *n* = 95; Mean ± SEM: 43.87% ± 2.758%. Females: *n* = 60; Mean ± SEM: 59.24% ± 3.701%) (Figure 1C), the LC difference increased to 30,861 cells/µL (Males: *n* = 97; Mean ± SEM: 45,581 ± 8,089. Females: *n* = 61; Mean ± SEM: 76,442 ± 16,062) (Figure 1D).

**Figure 2:**
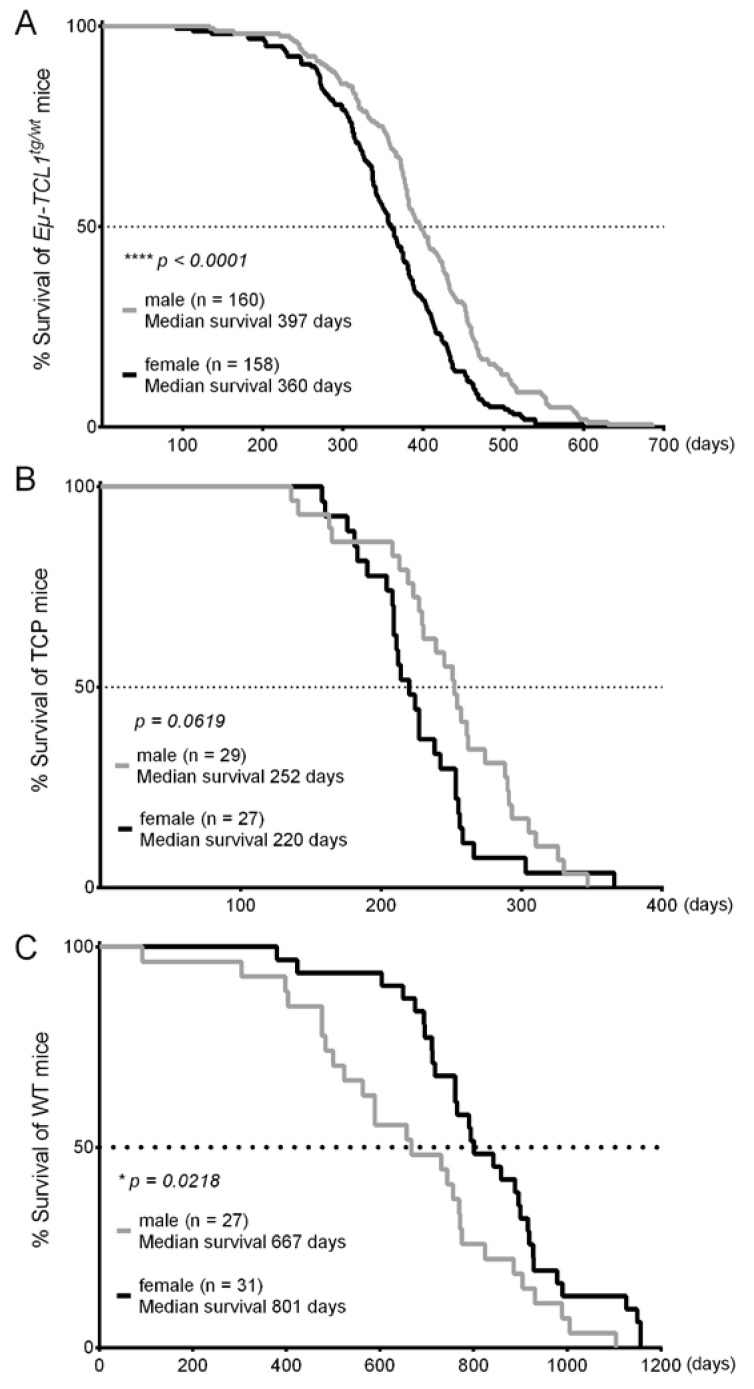
Significant sex difference in the overall survival of *Eµ-TCL1^tg/wt^* and *Eµ-TCL1^tg/wt^; CD19Cre^Cre/wt^; Trp53^fl/fl^* (TCP) mice. (**A**) Kaplan–Meier curves representing the overall survival of 160 male and 158 female *Eµ-TCL1^tg/wt^* mice showed significantly longer survival of males than females. Median survival: *Eµ-TCL1^tg/wt^* males = 397 days; *Eµ-TCL1^tg/wt^* females = 360 days. Mantel–Cox logrank test: **** *p* < 0.0001. (**B**) Kaplan–Meier curves representing the overall survival of 29 male and 27 female TCP mice demonstrating the longer survival of males than females. Median survival: TCP males = 252 days; TCP females = 220 days; Mantel–Cox logrank test: *p* = 0.0619. (**C**) Kaplan–Meier curves representing the overall survival of 27 male and 31 female wild type (WT) mice showed significantly longer survival of females compared to males, in contrast to the sex difference observed in the *Eµ-TCL1* transgenic mice. Median survival: WT males = 667 days, WT females = 801 days; Mantel–Cox logrank test: * *p* = 0.0218.

The substantial reduction in the number of females at 12 months as a consequence of their shorter OS also indicates the more progressive leukemic courses in females. In 10 mouse cohorts that were characterized independently by different investigators, a longer OS of the males could be congruently observed, independently of the mouse genetic background and housing facility (Table 1). Altogether, males (*n* = 160) had a median OS of 397 days, whereas the median OS of females (*n* = 158) was only 360 days, implying a significantly longer OS of 37 days in males than females (Figure 2A). Recently, the *Eµ-TCL1* transgenic mice have been crossed with other mouse lines lacking tumor-suppressor genes [15] or harboring an additional oncogene [16], leading to accelerated leukemia development and enhanced disease aggressiveness in these mice. To investigate whether these additional transgenic alleles could compromise the male–female bias in the *Eµ-TCL1* transgenic mice, we analyzed the OS of *Eµ-TCL1^tg/wt^; CD19Cre^Cre/wt^; Trp53^fl/fl^* (TCP) mice—a mouse model with features of high-risk human CLL. Due to B cell-specific deletion of *Tp53*, the TCP mice showed earlier disease onset, accelerated disease progression with occasional Richter transformation, and a significantly shorter lifespan than *Eµ-TCL1^tg/wt^* mice [15]. Despite a smaller cohort of TCP mice in our analysis, a clear trend in sex difference could also be observed. Independent of their genetic background and housing condition (Table 2), TCP males had a survival benefit of 32 days compared to TCP female littermates (Males: *n* = 29, median OS: 252 days, Females: *n* = 27, median OS: 220 days) (Figure 2B). This result suggests that the sex difference in leukemia development might be passed on to other mouse models with additional genetic lesions that were crossbred with the *Eµ-TCL1* transgenic mice. Of note, the shorter survival in females appeared to be a sole effect of *TCL1*-induced CLL in mice, because wildtype (WT) females lived longer than WT males in the same animal husbandry (Figure 2C). These WT mice are littermates of the *Eµ-TCL1^tg/wt^* mice in cohort #3 and #5 (Table 1).

**Figure 3:**
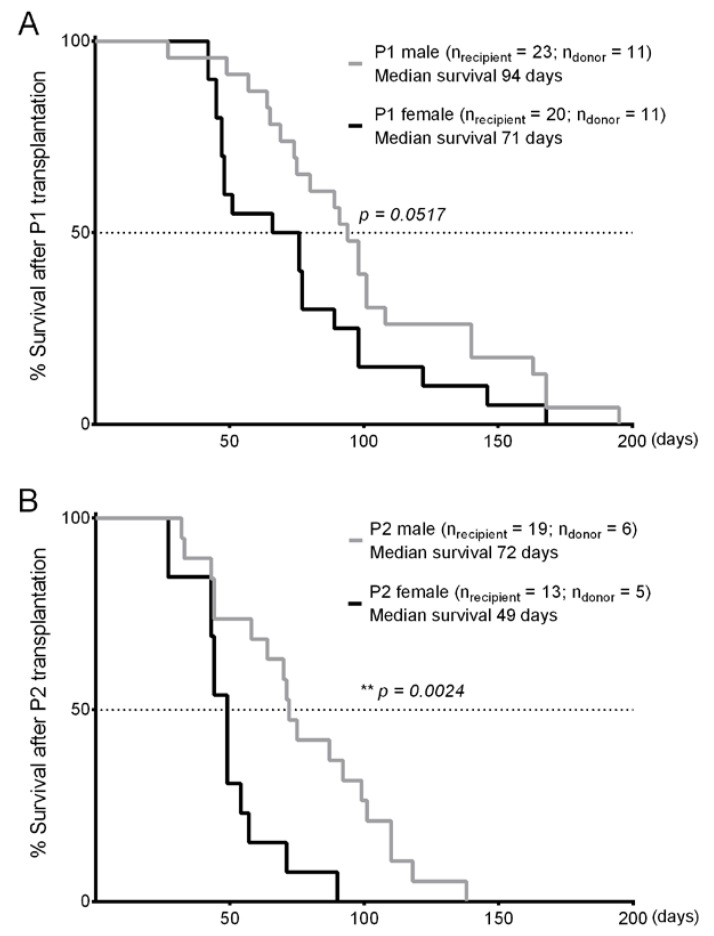
Significant sex difference in the survival of recipients after chronic lymphocytic leukemia (CLL) transplantation. (**A**) Kaplan–Meier curves of Passage 1 (P1) wild type (WT) recipient mice after syngeneic transplantation with murine CLL cells showing longer survival of males than females. Eleven male donor clones were transplanted in 23 male recipients; Eleven female clones were transplanted in 20 female recipients. Median survival: P1 males = 94 days, P1 females = 71 days; Mantel-Cox logrank test: *p* = 0.0517. (**B**) Kaplan–Meier curves of Passage 2 (P2) WT recipient mice after serial syngeneic transplantation with murine CLL cells showing longer survival of males than females. Six P1 male clones were transplanted in 19 male recipients; Five P1 female clones were transplanted in 13 female recipients. Median survival: P2 males = 72 days, P2 females = 49 days; Mantel–Cox logrank test: ** *p* = 0.0024.

Recently, the adoptive transplantation of murine CLL cells in immunocompetent mice, which significantly accelerates the CLL course in recipients compared to the *Eµ-TCL1* transgenic mice, is increasingly used, particularly in studies involving the CLL tumor microenvironment [14,17,18,19]. To determine if the sex difference in *Eµ-TCL1^tg/wt^* mice can also be observed in the adoptive transfer setting, we performed sequential transplantation of *Eµ-TCL1^tg/wt^* leukemia cells in syngeneic, immunocompetent recipient mice. Using strictly syngeneic donor and recipient mice of the C57Bl/6 (J) genetic substrain, 10^7^ CLL cells from each leukemic donor were injected peritoneally into sex-matched WT recipients. Here, Passage 1 (P1) male recipients lived 23 days longer than P1 females (Figure 3A), suggesting a tendency of sex disparity in the transplantation model. In a second transfer into Passage 2 (P2) recipients, a longer survival of 23 days could also be observed in P2 males compared to P2 females (Figure 3B), which represents a significant difference due to the shortened survival of P2 recipients compared to P1 recipients. In contrary to the sex-matched transplantation that always ensured engraftment and CLL progression in recipient mice, some sex-mismatched transplantation of leukemia cells failed to induce CLL, highlighting the importance of a sex-matched environment for CLL cells to engraft. In a few cases where CLL cells could be detected in sex-mismatched recipients, mismatched recipients had delayed disease onset and longer survival than sex-matched recipients receiving the same CLL cell clones. Of note, both male and female leukemia cells could grow in sex-mismatched recipients, which allows the sole effect of an immune response against the H-Y antigen to be excluded.

## 4. Discussion

Our analyses of over 300 *Eµ-TCL1^tg/wt^* mice and 56 TCP mice, together with the serial adoptive transplantation experiment revealed a significantly accelerated CLL progression followed by shorter survival in females compared to males. Despite the variable factors that might interfere with data acquisition such as husbandry, handling, and mouse genetic substrain, this sex difference in CLL progression is highly consistent in our *Eµ-TCL1^tg/wt^* study cohorts. Over a decade of breeding the *Eµ-TCL1^tg/wt^* mice with WT mice, the hereditary pattern in our cohorts allow the X-linked inheritance of the *TCL1*-transgene to be excluded. Furthermore, the highly complex and heterogeneous genetic landscape of murine *TCL1* tumors was recently elucidated by whole exome sequencing, which did not disclose major abnormalities in the sex chromosomes [20]. Although a causal genetic variation in sex chromosomes cannot be entirely excluded at the moment, it seems more likely that our observed difference between male and female *Eµ-TCL1^tg/wt^* mice is an effect of autosomal variants or of sex-specific features such as hormones, metabolisms, or epigenetic alterations. These factors are not only distinctive between the sexes but also have critical influences on cancer susceptibility and tumor growth [21]. Interestingly, *TCL1* as the oncogenic driver of this CLL mouse model was shown to be regulated by estrogen [22,23], to be involved in critical cancer-related metabolic pathways including glycolysis [24,25], and to inhibit DNA methylations [26,27]. Moreover, the stark differences in the immune cell subsets [28] and immune responses [29] between male and female mice might also contribute to the unequal leukemic growth, particularly in a malignancy strongly dependent on the immune niche such as CLL [18,30]. Thus, further studies to compare the molecular signatures of leukemic and immune cells in males versus females, or analyses of CLL development in *Eµ-TCL1^tg/wt^* mice with hormone therapy might be helpful approaches to identify the determinant factors underlying this sex difference.

In particular, our observation revealed a discrepancy of sex influence on disease progression in the *Eµ-TCL1* transgenic mouse model compared to CLL patients. Men are not only twice as likely to develop CLL, but also have a worse prognosis than women [1]. Several studies have consistently reported that female CLL patients had more benign clinical courses including reduced incidence, superior 10-year survival, and better treatment response [31,32,33]. In CLL cases with unmutated *IGHV* genes that can be modelled in the *Eµ-TCL1* transgenic mice [20,34], female patients also showed significantly longer survival [32]. Although the explicit elements underlying the gender disparity in human CLL still remain largely unknown, the levels of circulating sex hormones or altered DNA methylation in sex-related gene promoters have been suggested to be relevant factors contributing to the better prognosis of women [35,36].

This sharp contradiction to the human condition in the *Eµ-TCL1* transgenic mice might represent a drawback in modelling CLL and requires further investigations. Meanwhile, this sex difference should be promptly addressed during experimental design, data interpretation, and publications. First and foremost, comparisons between a male-excessive and a female-excessive cohort must be avoided [37]. Moreover, the generation of additional CLL models should be facilitated [38,39], with consideration to any possible disparity in CLL development between the sexes.

## 5. Conclusions

The use of both sexes and of sex-matched animal cohorts has been implemented in the standard procedure for animal studies [40] and should be reported transparently and precisely in publications. However, the number of male and female mice was only specified in a very limited number of papers involving the *Eμ-TCL1* transgenic mice, most likely due to unawareness of the significant sex difference in this model. Based on the results of this study, together with the report on the importance of the genetic background of *Eµ-TCL1* transgenic mice in the adoptive transplantation setting [41], we urge for more transparent, accurate descriptions of animal models in future publications, including specification of the male-to-female ratio in each study cohort and the accurate genetic substrains of the mice.

## Figures and Tables

**Table 1:** Characteristics of the analyzed *Eµ-TCL1^tg/wt^* mice.

No.	Ref.	Genetic Background	Additional, Unaffecting Transgene	Housing Facility	Number of Mice			Median Survival (Days)		
					Total	Male	Female	Male	Female	P (Log-Rank)
1	[12]	B6C3	-	EM	41	17	24	378	339.5	0.0080
2	[13]	B6C3	-	EM	36	15	21	424	369	0.0808
3	[14]	B6C3	-	EM	38	16	22	454.5	376.5	0.0025
4	[15]	B6 (J/N-mix)	Hemizygote CD19-Cre transgene without any loxP-flanked allele	PA	13	8	5	369	320	0.9621
5	-	B6 (J/N-mix)	LoxP-flanked mutant alleles without any Cre transgene	EM	29	17	12	402	358	0.2971
6	-	B6C3	-	EM	43	21	22	425	404.5	0.2700
7	-	B6C3	LoxP-flanked mutant alleles without any Cre transgene	EM	6	3	3	430	434	0.6537
8	-	B6C3	LoxP-flanked mutant alleles without any Cre transgene	EM	22	10	12	429.5	400.5	0.5522
9	-	B6C3	-	EM	47	28	19	357	345	0.2092
10	-	B6 (J)	-	CE	21	10	11	443	389	0.0466
11	-	B6 (J/N-mix)	-	PA	22	15	7	360	308	0.0079

**Table 2:** Characteristics of the analyzed *Eµ-TCL1^tg/wt^; CD19Cre^Cre/wt^; Trp53^fl/fl^* (TCP) mice.

No.	Ref.	Genetic Background	Housing Facility	Number of Mice			Median Survival (Days)		
				Total	Male	Female	Male	Female	P (Log-Rank)
1	[15]	B6 (J/N-mix)	PA	21	13	8	252	205.98	0.0223
2	-	B6 (J)	CE	35	16	19	256.5	227	0.2633

## References

[1] Hallek M., Shanafelt T.D., Eichhorst B. (2018). Chronic lymphocytic leukaemia. Lancet.

[2] Hallek M. (2019). Chronic lymphocytic leukemia: 2020 update on diagnosis, risk stratification and treatment. Am. J. Hematol..

[3] Bichi R., Shinton S.A., Martin E.S., Koval A., Calin G.A., Cesari R., Russo G., Hardy R.R., Croce C.M. (2002). Human chronic lymphocytic leukemia modeled in mouse by targeted tcl1 expression. Proc. Natl. Acad. Sci. USA.

[4] Johnson A.J., Lucas D.M., Muthusamy N., Smith L.L., Edwards R.B., De Lay M.D., Croce C.M., Grever M.R., Byrd J.C. (2006). Characterization of the tcl-1 transgenic mouse as a preclinical drug development tool for human chronic lymphocytic leukemia. Blood.

[5] Pekarsky Y., Drusco A., Kumchala P., Croce C.M., Zanesi N. (2015). The long journey of tcl1 transgenic mice: Lessons learned in the last 15 years. Gene Expr. J. Liver Res..

[6] Simonetti G., Bertilaccio M.T., Ghia P., Klein U. (2014). Mouse models in the study of chronic lymphocytic leukemia pathogenesis and therapy. Blood.

[7] Bresin A., D’Abundo L., Narducci M.G., Fiorenza M.T., Croce C.M., Negrini M., Russo G. (2016). Tcl1 transgenic mouse model as a tool for the study of therapeutic targets and microenvironment in human B-cell chronic lymphocytic leukemia. Cell Death Dis..

[8] Karp N.A., Mason J., Beaudet A.L., Benjamini Y., Bower L., Braun R.E., Brown S.D.M., Chesler E.J., Dickinson M.E., Flenniken A.M. (2017). Prevalence of sexual dimorphism in mammalian phenotypic traits. Nat. Commun..

[9] Zheng S., El-Naggar A.K., Kim E.S., Kurie J.M., Lozano G. (2007). A genetic mouse model for metastatic lung cancer with gender differences in survival. Oncogene.

[10] Naugler W.E., Sakurai T., Kim S., Maeda S., Kim K., Elsharkawy A.M., Karin M. (2007). Gender disparity in liver cancer due to sex differences in myd88-dependent il-6 production. Science.

[11] Zhai Y., Haresi A.J., Huang L., Lang D. (2020). Differences in tumor initiation and progression of melanoma in the braf(ca);tyr-creert2;pten(f/f) model between male and female mice. Pigment Cell Melanoma Res..

[12] Fedorchenko O., Stiefelhagen M., Peer-Zada A.A., Barthel R., Mayer P., Eckei L., Breuer A., Crispatzu G., Rosen N., Landwehr T. (2013). Cd44 regulates the apoptotic response and promotes disease development in chronic lymphocytic leukemia. Blood.

[13] Reinart N., Nguyen P.H., Boucas J., Rosen N., Kvasnicka H.M., Heukamp L., Rudolph C., Ristovska V., Velmans T., Mueller C. (2013). Delayed development of chronic lymphocytic leukemia in the absence of macrophage migration inhibitory factor. Blood.

[14] Nguyen P.H., Fedorchenko O., Rosen N., Koch M., Barthel R., Winarski T., Florin A., Wunderlich F.T., Reinart N., Hallek M. (2016). Lyn kinase in the tumor microenvironment is essential for the progression of chronic lymphocytic leukemia. Cancer Cell.

[15] Knittel G., Rehkamper T., Korovkina D., Liedgens P., Fritz C., Torgovnick A., Al-Baldawi Y., Al-Maarri M., Cun Y., Fedorchenko O. (2017). Two mouse models reveal an actionable parp1 dependence in aggressive chronic lymphocytic leukemia. Nat. Commun..

[16] Lucas F., Rogers K.A., Harrington B.K., Pan A., Yu L., Breitbach J., Bundschuh R., Goettl V.M., Hing Z.A., Kanga P. (2019). Emu-tcl1xmyc: A novel mouse model for concurrent cll and B-cell lymphoma. Clin. Cancer Res..

[17] Lutzny G., Kocher T., Schmidt-Supprian M., Rudelius M., Klein-Hitpass L., Finch A.J., Durig J., Wagner M., Haferlach C., Kohlmann A. (2013). Protein kinase c-beta-dependent activation of nf-kappab in stromal cells is indispensable for the survival of chronic lymphocytic leukemia B cells in vivo. Cancer Cell.

[18] Nguyen P.H., Niesen E., Hallek M. (2019). New roles for B cell receptor associated kinases: When the B cell is not the target. Leukemia.

[19] Dong S., Harrington B.K., Hu E.Y., Greene J.T., Lehman A.M., Tran M., Wasmuth R.L., Long M., Muthusamy N., Brown J.R. (2019). Pi3k p110delta inactivation antagonizes chronic lymphocytic leukemia and reverses T cell immune suppression. J. Clin. Investig..

[20] Zaborsky N., Gassner F.J., Hopner J.P., Schubert M., Hebenstreit D., Stark R., Asslaber D., Steiner M., Geisberger R., Greil R. (2019). Exome sequencing of the tcl1 mouse model for cll reveals genetic heterogeneity and dynamics during disease development. Leukemia.

[21] Cheng F. (2016). Gender dimorphism creates divergent cancer susceptibilities. Trends Cancer.

[22] Ho M.F., Bongartz T., Liu M., Kalari K.R., Goss P.E., Shepherd L.E., Goetz M.P., Kubo M., Ingle J.N., Wang L. (2016). Estrogen, snp-dependent chemokine expression and selective estrogen receptor modulator regulation. Mol. Endocrinol..

[23] Badve S., Collins N.R., Bhat-Nakshatri P., Turbin D., Leung S., Thorat M., Dunn S.E., Geistlinger T.R., Carroll J.S., Brown M. (2010). Subcellular localization of activated akt in estrogen receptor- and progesterone receptor-expressing breast cancers: Potential clinical implications. Am. J. Pathol..

[24] Nishimura K., Aizawa S., Nugroho F.L., Shiomitsu E., Tran Y.T.H., Bui P.L., Borisova E., Sakuragi Y., Takada H., Kurisaki A. (2017). A role for klf4 in promoting the metabolic shift via tcl1 during induced pluripotent stem cell generation. Stem Cell Rep..

[25] Fiorenza M.T., Rava A. (2019). The tcl1 function revisited focusing on metabolic requirements of stemness. Cell Cycle.

[26] Chen S.S., Raval A., Johnson A.J., Hertlein E., Liu T.H., Jin V.X., Sherman M.H., Liu S.J., Dawson D.W., Williams K.E. (2009). Epigenetic changes during disease progression in a murine model of human chronic lymphocytic leukemia. Proc. Natl. Acad. Sci. USA.

[27] Palamarchuk A., Yan P.S., Zanesi N., Wang L., Rodrigues B., Murphy M., Balatti V., Bottoni A., Nazaryan N., Alder H. (2012). Tcl1 protein functions as an inhibitor of de novo DNA methylation in B-cell chronic lymphocytic leukemia (cll). Proc. Natl. Acad. Sci. USA.

[28] Hensel J.A., Khattar V., Ashton R., Ponnazhagan S. (2019). Characterization of immune cell subtypes in three commonly used mouse strains reveals gender and strain-specific variations. Lab. Investig..

[29] Lin P.Y., Sun L., Thibodeaux S.R., Ludwig S.M., Vadlamudi R.K., Hurez V.J., Bahar R., Kious M.J., Livi C.B., Wall S.R. (2010). B7-h1-dependent sex-related differences in tumor immunity and immunotherapy responses. J. Immunol..

[30] Burger J.A., Wiestner A. (2018). Targeting B cell receptor signalling in cancer: Preclinical and clinical advances. Nat. Rev. Cancer.

[31] Molica S. (2006). Sex differences in incidence and outcome of chronic lymphocytic leukemia patients. Leuk. Lymphoma.

[32] Catovsky D., Wade R., Else M. (2014). The clinical significance of patients’ sex in chronic lymphocytic leukemia. Haematologica.

[33] Mauro F.R., Foa R., Giannarelli D., Cordone I., Crescenzi S., Pescarmona E., Sala R., Cerretti R., Mandelli F. (1999). Clinical characteristics and outcome of young chronic lymphocytic leukemia patients: A single institution study of 204 cases. Blood.

[34] Yan X.J., Albesiano E., Zanesi N., Yancopoulos S., Sawyer A., Romano E., Petlickovski A., Efremov D.G., Croce C.M., Chiorazzi N. (2006). B cell receptors in tcl1 transgenic mice resemble those of aggressive, treatment-resistant human chronic lymphocytic leukemia. Proc. Natl. Acad. Sci. USA.

[35] Allain E.P., Venzl K., Caron P., Turcotte V., Simonyan D., Gruber M., Le T., Lévesque E., Guillemette C., Vanura K. (2018). Sex-dependent association of circulating sex steroids and pituitary hormones with treatment-free survival in chronic lymphocytic leukemia patients. Ann. Hematol..

[36] Lin S., Liu Y., Goldin L.R., Lyu C., Kong X., Zhang Y., Caporaso N.E., Xiang S., Gao Y. (2019). Sex-related DNA methylation differences in B cell chronic lymphocytic leukemia. Biol. Sex Differ..

[37] Shansky R.M. (2019). Are hormones a "female problem" for animal research?. Science.

[38] Klein U., Lia M., Crespo M., Siegel R., Shen Q., Mo T., Ambesi-Impiombato A., Califano A., Migliazza A., Bhagat G. (2010). The dleu2/mir-15a/16-1 cluster controls B cell proliferation and its deletion leads to chronic lymphocytic leukemia. Cancer Cell.

[39] Yin S., Gambe R.G., Sun J., Martinez A.Z., Cartun Z.J., Regis F.F.D., Wan Y., Fan J., Brooks A.N., Herman S.E.M. (2019). A murine model of chronic lymphocytic leukemia based on B cell-restricted expression of sf3b1 mutation and atm deletion. Cancer Cell.

[40] Clayton J.A., Collins F.S. (2014). Policy: Nih to balance sex in cell and animal studies. Nature.

[41] Ozturk S., Roessner P.M., Schulze-Edinghausen L., Yazdanparast H., Kalter V., Lichter P., Hanna B.S., Seiffert M. (2019). Rejection of adoptively transferred emicro-tcl1 chronic lymphocytic leukemia cells in c57bl/6 substrains or knockout mouse lines. Leukemia.

